# Slow Electron Making More Efficient Radiation Emission

**DOI:** 10.1038/s41598-018-23203-x

**Published:** 2018-03-20

**Authors:** Dong-Sing Wuu, Sin-Liang Ou, Ching-Ho Tien

**Affiliations:** 1 Department of Materials Science and Engineering, National Chung Hsing University, Taichung, 40227 Taiwan, R.O.C.; 2 Department of Materials Science and Engineering, Da-Yeh University, Changhua, 51591 Taiwan, R.O.C.

## Abstract

In conventional emitting devices, the mobility of electron is much higher than that of hole, which increases the non-recombination rate. To generate slow electrons, we demonstrate an electron retarding n-electrode (ERN) on the n-GaN layer of InGaN blue light emitting diode (LED), making more efficient radiation emission. Transparent conductive oxides are estimated to be more suitable for ERN materials. However, for ERN materials used in InGaN LEDs, three requirements should be satisfied, i.e., Ohmic contact to n-GaN, dilute magnetic doping, and good electrical conductivity. The pulsed-laser deposited cobalt-doped ZnO film prepared at 400 °C was chosen as the ERN. The electron retarding of 120-nm-thick ERN/n-GaN reached 19.9% compared to the n-GaN. The output powers (@350 mA) of LEDs with and without the ERN were 246.7 and 212.9 mW, while their wall-plug efficiencies were 18.2% and 15.1%, respectively. Moreover, owing to the efficient filling of electrons in the quantum wells by inserting the ERN, the bandgap of quantum wells was enlarged, inducing the blue-shift in the emission wavelength of LED. The slow electron generated from the ERN technique paves the way to solve the problem of large difference between electron and hole velocities and improve the optoelectronic performance of emitting devices.

## Introduction

Recently, with the invention of emitting devices, such as laser diodes (LDs) and light emitting diodes (LEDs), the human life becomes more convenient^1–3^. Moreover, the improvement in the efficiency of emitting devices can expand their practicalities. Up to now, to enhance the emission efficiency, several issues consisting of the light extraction, optical design, heat dissipation, package technique, carrier confinement, and internal quantum efficiency (IQE) have been solved for the emitting devices^4–9^. Even though the emission efficiency of devices can be improved efficiently, an essential problem in these emitting devices, i.e., the excessively large velocity (mobility) difference between electron and hole carriers, is still not overcome. For example, the velocity of electron in a conventional blue InGaN LED is approximately 26 times faster than that of hole. This large difference between electron and hole velocities would increase the non-recombination rate of the device, degrading its emission performance^10,11^. Actually, to increase the recombination rate of electron-hole pairs of InGaN LEDs, the electron blocking layer (EBL)^12^ and electron tunneling barrier (ETB)^13^ were incorporated into the epitaxial structures. However, the hole blocking problem and the increment of defect density in the multiple quantum well (MQW) region could be generated as the EBL and ETB were used, respectively. Most importantly, the velocity of electron in the LED is not affected through the insertions of EBL and ETB. Thus, how to create the electrons with the slower velocity in the emitting devices becomes a very interesting challenge.

**Figure 1:**
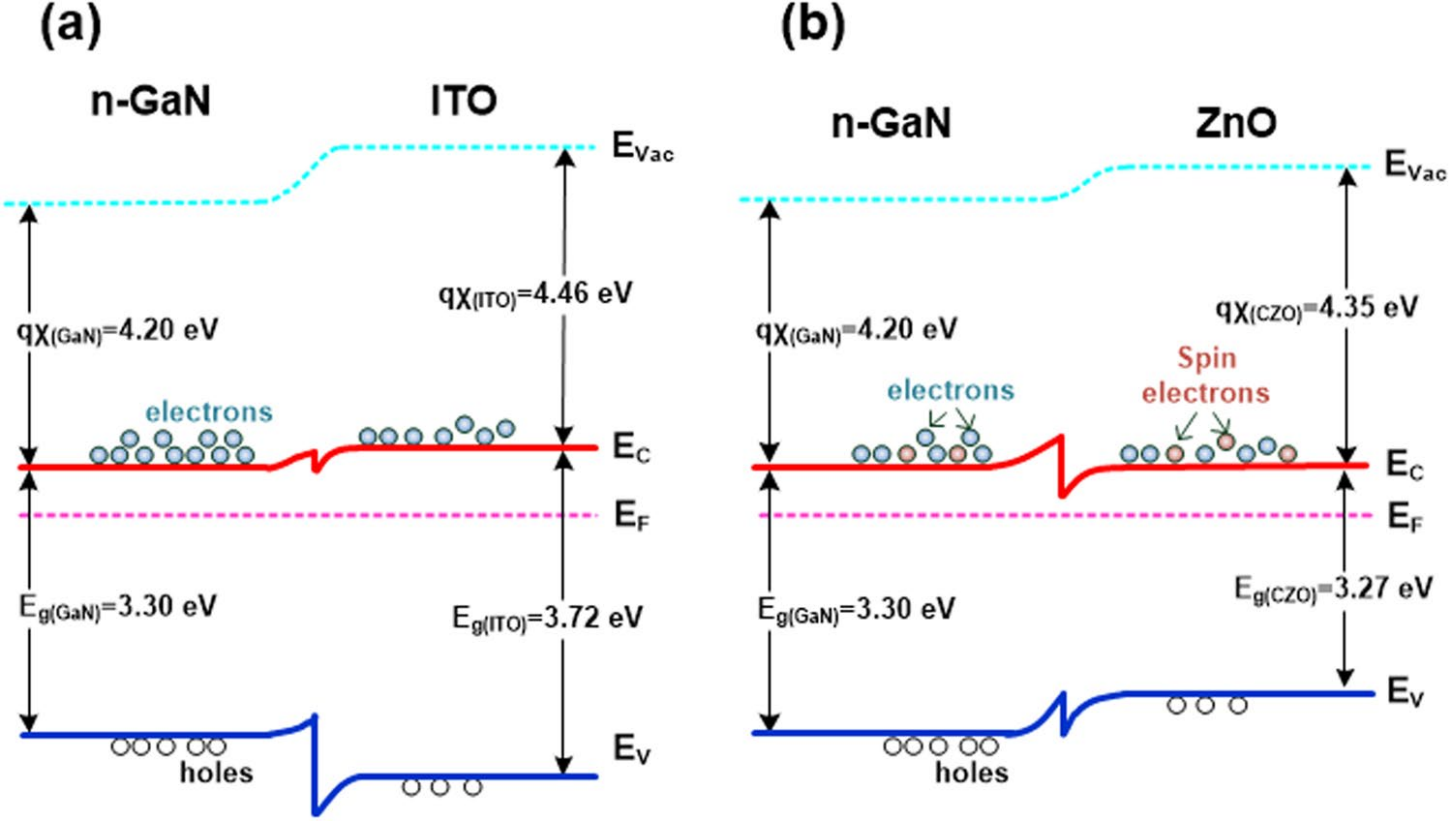
Band diagrams of (**a**) ITO/n-GaN and (**b**) ZnO/n-GaN isotype heterojunctions.

In this research, to produce the slow electrons in the emitting device, we have proposed the electron retarding n-electrode (ERN) on the n-GaN layer to enhance the optoelectronic performance of nitride LEDs. In conventional LED devices, metal electrodes (such as Ti/Al) are usually directly prepared on n-GaN; however, most of metals are not good candidates as the ERNs. This is because the electron mobilities are very high in most of metals. Transparent conductive oxides (TCOs) are the possible candidates as the ERNs. At present, very few researches on the TCO/n-GaN have been presented. ITO and ZnO are more often prepared on n-GaN layers. Figure 1a,b shows the band diagrams of ITO/n-GaN and ZnO/n-GaN isotype heterojunctions, respectively^14–17^. In comparison to the Ohmic contact between ZnO and n-GaN, ITO usually has the Schottky contact with the n-GaN. This is attributed to the barrier heights of 0.63–0.95 eV between ITO and n-GaN^14,15^. Thus, ZnO-based materials could be more feasible as the ERNs than ITO.

The characteristics of various TCOs (consisting of ITO, ZnO, ITO-ZnO, Ga_2_O_3_, and Co-doped ZnO) prepared on n-GaN were summarized in Table 1^14–16,18,19^. Even though the contact characteristic between ITO and n-GaN can be transferred from Schottky to Ohmic, ITO is still not a good ERN. A promising ERN (on n-GaN) should satisfy these three requirements simultaneously. First, the contact characteristic between ERN and n-GaN is Ohmic, ensuring the LEDs can be operated. Second, the ERN should be incorporated with small amount magnetic ions (i.e., the dilute magnetic doping)^20^. This is attributed that the electrons can be scattered because of the collisions between the magnetic ions (spin-orbit interaction) and the electrons, resulting in the decrease of the electron mobility. Is the TCO with the heavy magnetic doping also a suitable ERN? This will result in the formation of magnetic metal-oxides, which are not help to scatter the electrons and could degrade its conductivity. Finally, the ERN with a good electrical conductivity is also required. When the ERN has a good electrical conductivity, a more uniform current injection can be achieved in the LED with inserting the ERN, which induces a higher probability of encountering between electrons and magnetics ions. As shown in Table 1, ITO, ZnO, ITO-ZnO, and Ga_2_O_3_ all are not suitable to be ERNs on n-GaN. In our study, the cobalt-doped ZnO (Co-doped ZnO, CZO) film prepared by pulsed laser deposition (PLD) can simultaneously satisfy these three requirements, and it was verified to be a promising ERN. Via the deposition of CZO-ERN on n-GaN, the electron mobility becomes slower, making more efficient radiation emission on nitride LEDs. Most importantly, the problem of the excessively large velocity difference between electron and hole carriers occurred in various emitting devices can be successfully solved by introducing the slow electrons.

**Table 1:** A summary of various TCOs prepared on n-GaN. The contact to n-GaN, resistivity, dilute magnetic doping, and electron retarding properties of TCOs are compared.

Material	Method	Contact to n-GaN	Resistivity (Ω-cm)	Dilute magnetic doping	Electron retarding	Ref.
ITO	evaporation	Schottky	~10^−4^	w/o	w/o	^14^
ITO	sputtering & annealing	Ohmic	~10^−4^	w/o	w/o	^15^
ZnO:Ga	chemical vapor de position	Ohmic	~10^−1^–10^−2^	w/o	w/o	^16^
ITO-ZnO	co-sputtering	Schottky	3.82 × 10^−4^	w/o	w/o	^18^
Ga_2_O_3_	PLD	Schottky	>10^5^	w/o	w/o	^19^
Co-doped ZnO	PLD	Ohmic	4.3 × 10^−2^	w	w	Our study

## Results and Discussion

**Figure 2:**
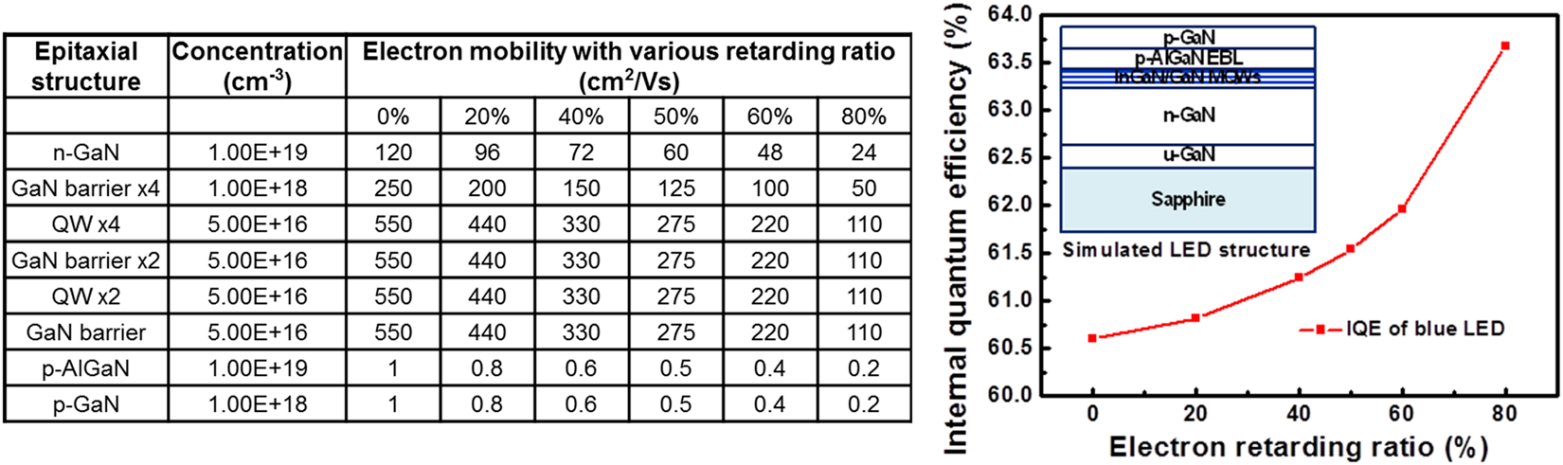
Details of the simulated epitaxial structure for the blue LED (including concentrations and mobilities of various epitaxial layers) and the simulated IQE performance with various electron retarding ratios.

To investigate the effect of slow electron on the emission property of nitride LED, the internal quantum efficiencies (IQEs) of the conventional blue LED as a function of the electron retarding ratio were simulated using SiLENSe software. The electron retarding ratio is defined as $\frac{{\mu }_{a}-{\mu }_{b}}{{\mu }_{a}}$, where μ_a_ and μ_b_ are the original and reduced mobilities of n-GaN, respectively. Figure 2 shows the details of the simulated epitaxial structure for the blue LED (including concentrations and mobilities of various epitaxial layers) and the simulated IQE performance with various electron retarding ratios (from 0% to 80%). Based on the simulated results, the IQE of blue LED can be increased from 60.6% to 63.67% with increasing the electron retarding ratio from 0% to 80%.

**Figure 3:**
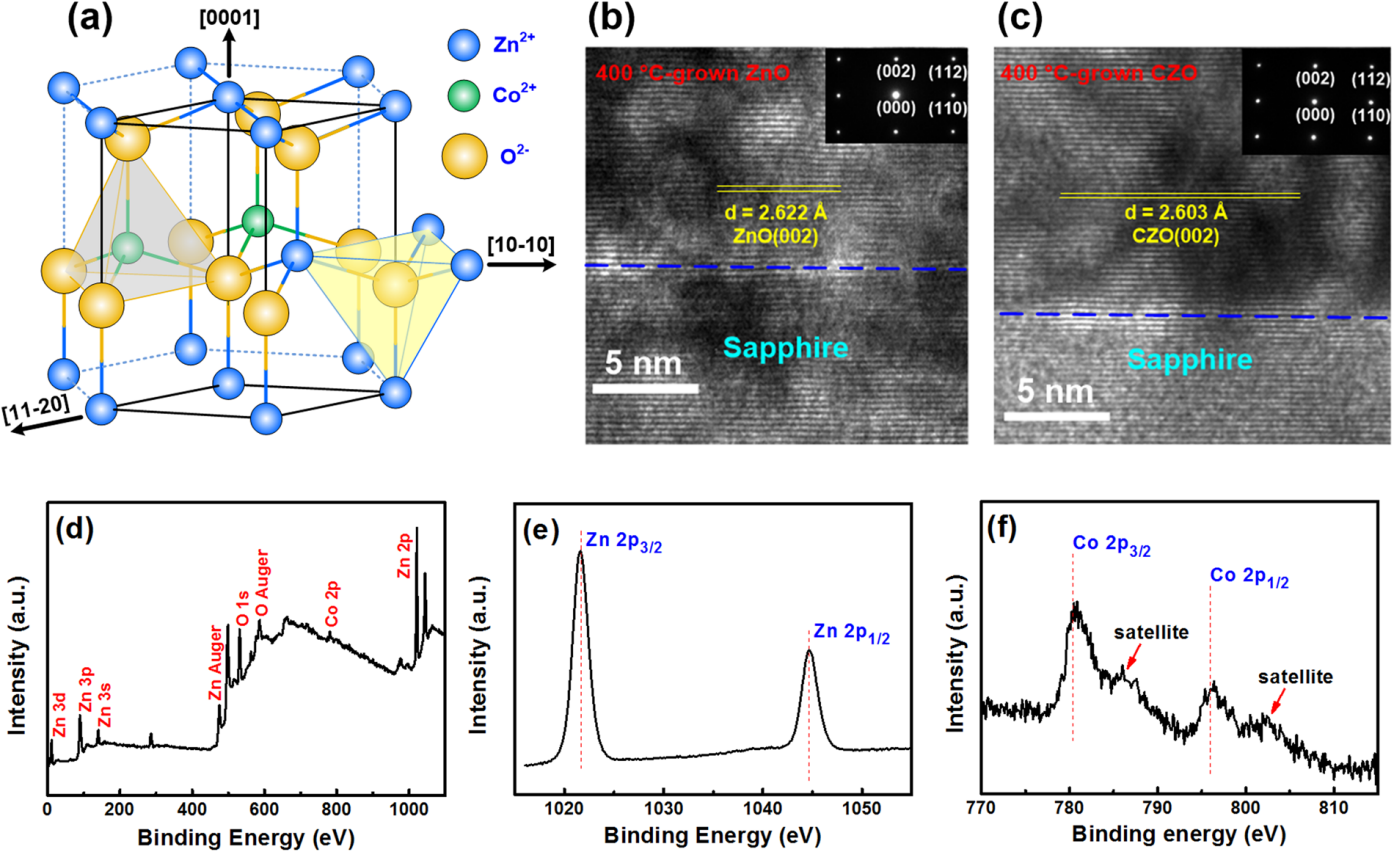
(**a**) Schematic of the crystal structure of the CZO material. High resolution TEM images and selected area electron diffraction patterns of 400 °C-grown (**b**) ZnO and (**c**) CZO films. (**d**) XPS survey scan spectrum (**e**) high-resolution Zn 2p core level XPS spectrum, and (**f**) high-resolution Co 2p core level XPS spectrum of 400 °C-grown CZO film.

As mentioned above, the dilute-magnetic doped ZnO (Ohmic contact with n-GaN and good electrical conductivity) could be the promising ERN for InGaN LEDs. In comparison to other magnetic elements, Co metal is a very suitable material as the dopant into ZnO because a remarkable magnetization per Co ion for very low substitutions can be obtained in the Co-doped sample. This is why we chose the CZO film as the ERN. Figure 3a shows the schematic of the crystal structure of the CZO material. The ion radii of Zn^2+^ and Co^2+^ are 74 and 70 Å, respectively. The ion radius of Co^2+^ is smaller than that of Zn^2+^. Thus, the substitutions of Zn^2+^ ions with Co^2+^ in the ZnO lattice will slightly reduce its d-spacing value. Figure 3b,c displays the high resolution TEM images and selected area electron diffraction patterns of ZnO and CZO films (both prepared at the T_s_ of 400 °C), respectively. In comparison to the d-spacing value of ZnO(002) (2.622 Å), the CZO film had the smaller d-spacing value (2.603 Å). Additionally, the X-ray photoelectron spectroscopy (XPS) measurement was also used to analyze the 400 °C-grown CZO film. The measured depth for the 120-nm-thick CZO film is about 60 nm. All the XPS peaks in the survey scan spectrum could be identified to Zn, O, and Co, as shown in Fig. 3d. We can find that no other impurities exist in the CZO film. Figure 3e displays the high-resolution Zn 2p core level XPS spectrum of the CZO film. Two peaks indexed as Zn 2p3/2 and Zn 2p1/2 centered at 1021.6 and 1044.6 eV, respectively. The binding energy difference (23.0 eV) between these two peaks is consistent with that of ZnO^21^. The high-resolution Co 2p core level XPS spectrum of the CZO film is exhibited in Fig. 3f. Two main peaks of Co 2p3/2 and Co 2p1/2 located at 780.5 and 796.0 eV, respectively. Moreover, two shake-up satellites at higher binding energy were also observed, which was considered as a feature of Co^2+^ ions^22^. The binding energy difference (15.5 eV) between Co 2p3/2 and Co 2p1/2 indicates that the Co^2+^ ions are formed in CoO and Co:ZnO^23^. This reveals that the Co^2+^ ions have successfully substituted for Zn^2+^ ions in the ZnO lattice. The Co, Zn, and O concentrations of this film were measured to be 2.7 at.%, 56.6 at.%, and 40.7 at.%, respectively. Thus, based on TEM and XPS results, it can be confirmed that the Co ions are indeed doped into the ZnO lattice.

**Figure 4:**
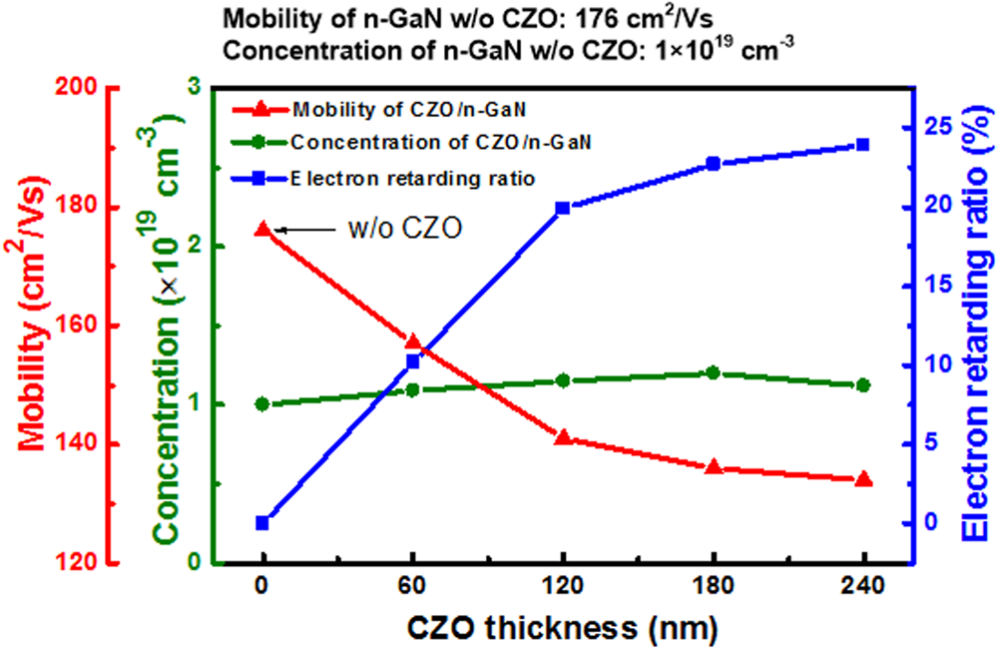
Mobilities, carrier concentrations, and retarding electron ratios of CZO/n-GaN samples as a function of CZO thickness from 0 to 240 nm.

Due to the homogeneous microstructure and random distribution of Co^2+^ ions in the CZO film, the probability of encountering between electrons and Co^2+^ ions can be increased with an increment of CZO thickness. This can lead to the more efficient electron scattering, improving its electron retarding effect. Figure 4 shows the mobilities, carrier concentrations, and retarding electron ratios of CZO/n-GaN samples as a function of CZO thickness from 0 to 240 nm. The detailed measurement method for electrical properties of CZO/n-GaN samples was described in our previous research^20^. The mobilities and carrier concentrations were both analyzed by Hall measurements. Without depositing the CZO film on n-GaN, the mobility and carrier concentration of n-GaN were 176 cm^2^/Vs and 1 × 10^19^ cm^−3^, respectively. It should be mentioned that the n-GaN sample (without depositing the CZO layer) represents the metal Ohmic contact on n-GaN. With increasing the CZO thickness to 240 nm, the mobility of CZO/n-GaN decreased to 134 cm^2^/Vs, while its electron retarding ratio increased to 23.9%. The phenomenon of mobility reduction occurred in the CZO/n-GaN samples (in comparison to the n-GaN sample) can be verified that the velocity of electron is slowed down efficiently via the insertion of the CZO layer. Although the mobility of CZO/n-GaN sample reduced gradually by increasing the CZO thickness, we can see that the reduction speed of mobility became obviously slow when the CZO thickness was increased to 120 nm. The mobility and electron retarding ratio of 120-nm-thick CZO/n-GaN sample were 141 cm^2^/Vs and 19.9%, respectively. This indicates the electron retarding effect becomes saturated as the CZO thickness is larger than 120 nm. However, it can be found the carrier concentrations of all CZO/n-GaN samples are similar to that of n-GaN, revealing the carrier concentration of n-GaN is not influenced by inserting the CZO film.

**Figure 5:**
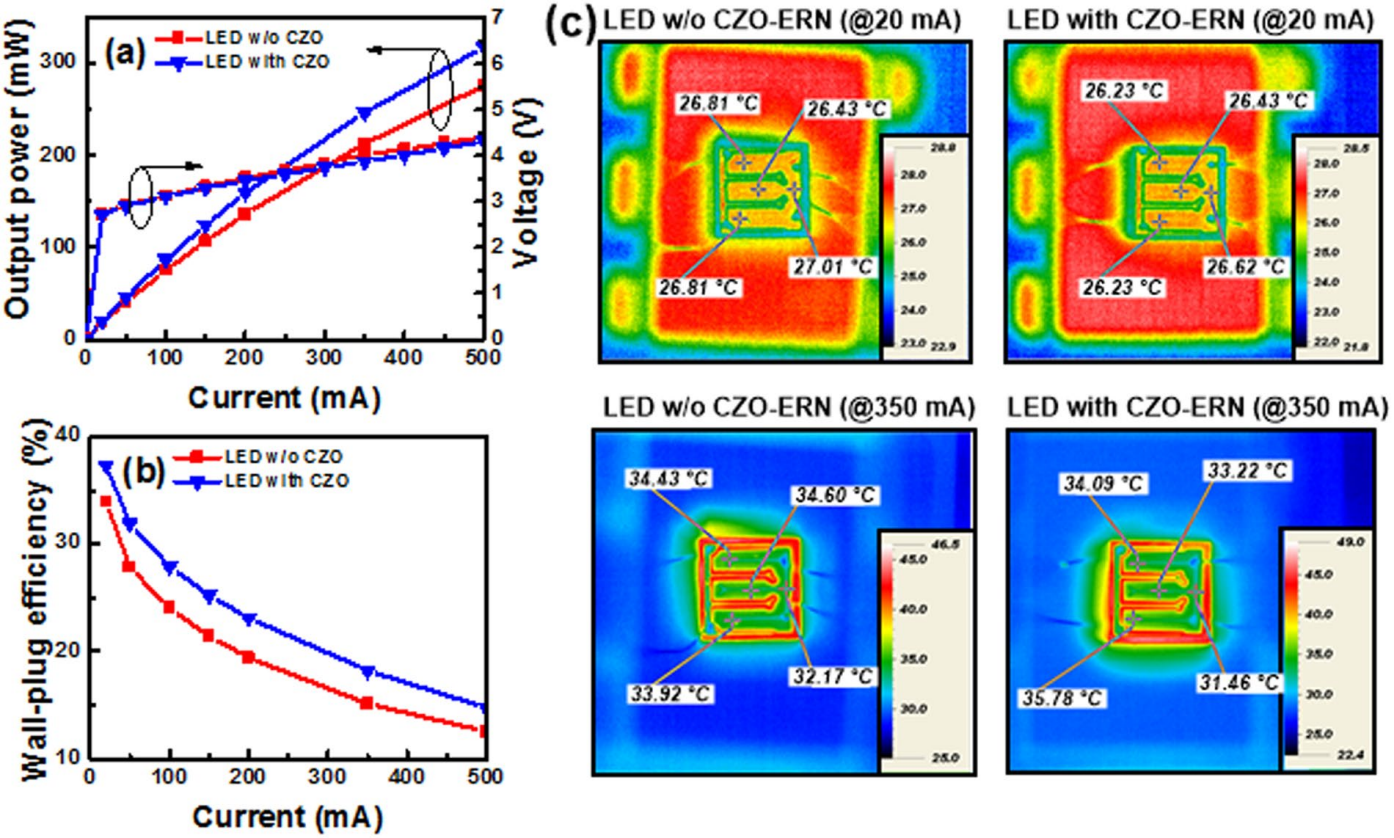
(**a**) Forward voltages, output powers and (**b**) wall-plug efficiencies as a function of the injection current for blue LEDs with and without inserting the ERN. (**c**) Surface temperature distributions of blue LEDs with and without inserting the ERN at 20 and 350 mA injection currents.

According to the results of Fig. 4, the 120-nm-thick CZO film was selected as the ERN to fabricate the blue LED. Figure 5a shows the forward voltages and output powers as a function of the injection current of blue LEDs with and without inserting the ERN. These two LEDs had almost the same forward voltages (@0-500 mA). Based on the results of Fig. 4 (carrier concentration of n-GaN) and 5a, we can confirm the electrical characteristics of the LED are not affected by inserting the ERN. As the devices were operated at 350 mA, the output powers of the blue LEDs with and without the ERN were 246.7 and 212.9 mW, respectively. Further increasing the injection current to 500 mA, the output powers of these two LEDs were measured to be 317.9 and 276.2 mW, respectively. After inserting the ERN into LED, 15.9% and 15.1% improvements can be achieved in the output powers (@350 and 500 mA) compared with those of conventional LED. Figure 5b shows the wall-plug efficiencies (WPEs) of these two LEDs versus the injection current. The WPE of the LED with the CZO-ERN is obviously higher than that of conventional LED. The WPEs of these two LEDs at 350 mA were 18.2% and 15.1%, respectively. Figure 5c displays the surface temperature distributions of LEDs with and without inserting the CZO-ERN at 20 and 350 mA injection currents. We can observe that the surface temperatures (@20 mA) of LEDs with and without the ERN are 26.23–26.62 and 26.43–27.01 °C, respectively. Further increasing the driving current to 350 mA, the surface temperatures of these two LEDs are increased to 31.48–35.78 and 32.17–34.60 °C, respectively. Obviously, the surface temperatures for these two LEDs are much similar to each other, indicating the thermal characteristic of blue LED cannot be affected via the addition of the ERN.

**Figure 6:**
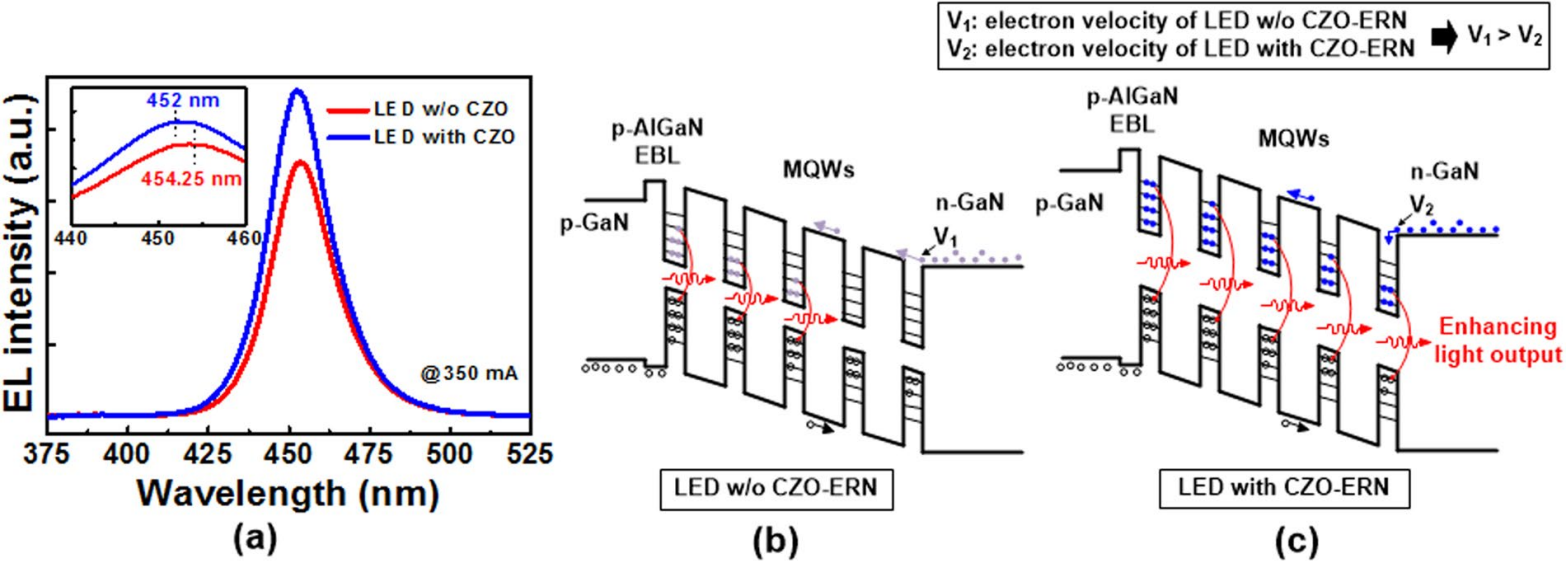
(**a**) EL spectra (@350 mA) of blue LEDs with and without the CZO-ERN. Carrier transport models of LEDs (**b**) without and (**c**) with CZO-ERN.

Except for the emission improvement after inserting the CZO-ERN, the other interesting phenomenon was also induced through the formation of slow electrons. Figure 6a shows the EL spectra (@350 mA) of blue LEDs with and without the CZO-ERN. Without inserting the ERN, the peak wavelength of EL emission was centered at 454.25 nm, as shown in the inset of Fig. 6a. After adding the ERN, the peak wavelength of EL emission shifted to the shorter wavelength of 452 nm (blue-shift). Additionally, the full width at half maximum (FWHM) value of the EL emission peak for the blue LED with the ERN was slightly larger than that without the ERN. To explain the EL results, the carrier transport models of LEDs without and with CZO-ERN were established, as shown in Fig. 6b and c, respectively. Without inserting the ERN, the velocity of electron is much faster than that of hole, and the electrons are difficult to fill each quantum well (Fig. 6b). This will lead to the gather of electrons in the quantum wells near the p-AlGaN EBL to radiatively recombine with holes. Thus, the non-radiative recombination rate in the MQWs is increased. However, as discussed above, the velocity of electron can be slowed down efficiently after inserting the ERN. Therefore, as shown in Fig. 6c, electrons are easier to fill the quantum wells, and electrons and holes can more uniformly distribute in the MQWs, enhancing the radiative recombination rate and increasing the FWHM value of the emission peak. On the other hand, because electrons are filled in the quantum wells more effectively by inserting the ERN, electrons can transit from a high energy level to a low energy level to recombine with holes. As a result, the bandgap of MQWs can be enlarged, leading to the blue-shift in the peak wavelength of EL emission.

## Conclusion

In summary, the slow electrons were successfully generated by depositing the CZO-ERN on the n-GaN layer, reducing the mobility difference between electron and hole carriers and making more efficient radiation emission of InGaN blue LEDs. Dilute magnetic TCOs with good electrical conductivity and Ohmic contact characteristic to n-GaN have great potential for ERN applications. After depositing the 120-nm-thick CZO-ERN on n-GaN, the slow electrons with the electron retarding ratio of 19.9% can be generated. In comparison to the LED without inserting the ERN, the LED with the ERN possessed 15.9% enhancement in the output power (@350 mA). Meanwhile, the WPEs (@350 mA) of LEDs with and without the ERN were 18.2% and 15.1%, respectively. This indicates the slow electrons formed via the ERN technique indeed can improve the recombination rate of electron-hole pairs. Additionally, based on the EL results, the peak wavelengths of emission for these two devices were centered at 454.25 and 452 nm, respectively. The blue-shift in emission wavelength occurred in the LED with inserting the ERN is attributed to the efficient filling of electrons in the quantum wells. The results reveal that the ERN technique is highly feasible for generating the slow electrons and enhancing the optoelectronic performance of emitting devices. Via the introduction of slow electrons, the excessively large velocity difference between electron and hole carriers occurred in various emitting devices can be reduced efficiently.

## Methods

The CZO films (thickness: 60–240 nm) were prepared by PLD at the substrate temperature (T_s_) of 400 °C in Ar atmosphere. During the CZO growth, a stoichiometric ceramic target with the composition of 95 at.% ZnO + 5 at.% Co was used. Detailed deposition conditions of CZO were presented in our previous study^24^. The CZO film deposited at 400 °C was used as the ERN due to its good electrical properties. To confirm the slow electron effect of CZO-ERN, lateral-type blue LEDs with and without CZO-ERN were both prepared on sapphire substrates by metalorganic chemical vapor deposition^20^. The LED structures comprised a 2-μm-thick u-GaN, a 2-μm-thick n-type GaN:Si, 6 pairs of InGaN/GaN MQW active layer, a 0.1-μm-thick p-type AlGaN, and a 0.4-μm-thick p-type GaN:Mg. The chip size and emission wavelength of the blue LED are 45 × 45 mil^2^ and 450 nm, respectively. For the device process, the 80-nm-thick ITO film was used as a transparent conductive layer on the p-GaN. The CZO-ERN was grown on the n-GaN before preparing the n-pad metal. Furthermore, the Cr/Au (20 nm/200 nm) was used as both n- and p-pads.

The lattice image of CZO film was observed by transmission electron microscopy (TEM). Chemical states and compositions of the CZO film were characterized by X-ray photoelectron spectroscopy (XPS, ULVAC-PHIPHI 5000). Electrical characteristics of CZO/n-GaN samples were investigated via the van der Pauw Hall measurements (ACCENT, HL-5500PC). The current-voltage (I-V) properties of LEDs were investigated by a semiconductor parameter analyzer (Keithley, 2400 sourcemeter), and the output powers were determined with a calibrated integrating sphere. The electroluminescence (EL) measurement at room temperature was used to analyze the emission properties of LEDs.
